# Mineralocorticoid receptor antagonism in diabetes reduces albuminuria by preserving the glomerular endothelial glycocalyx

**DOI:** 10.1172/jci.insight.154164

**Published:** 2023-03-08

**Authors:** Michael Crompton, Joanne K. Ferguson, Raina D. Ramnath, Karen L. Onions, Anna S. Ogier, Monica Gamez, Colin J. Down, Laura Skinner, Kitty H. Wong, Lauren K. Dixon, Judit Sutak, Steven J. Harper, Paola Pontrelli, Loreto Gesualdo, Hiddo L. Heerspink, Robert D. Toto, Gavin I. Welsh, Rebecca R. Foster, Simon C. Satchell, Matthew J. Butler

**Affiliations:** 1 Bristol Renal, Translational Health Sciences, Bristol Medical School, University of Bristol, Bristol, United Kingdom.; 2 Pathology Department, Southmead Hospital, Bristol, United Kingdom.; 3 School of Physiology, Pharmacology & Neuroscience, University of Bristol, Bristol, United Kingdom.; 4 Division of Nephrology, Dialysis and Transplantation, Department of Emergency and Organ Transplantation, Aldo Moro University of Bari, Bari, Italy.; 5 Department of Clinical Pharmacology, University Medical Center Groningen, University of Groningen, The Netherlands.; 6 Department of Clinical Sciences, The University of Texas Southwestern Medical Center, Dallas, Texas, USA.

**Keywords:** Endocrinology, Nephrology, Chronic kidney disease, Diabetes, Glycobiology

## Abstract

The glomerular endothelial glycocalyx (GEnGlx) forms the first part of the glomerular filtration barrier. Previously, we showed that mineralocorticoid receptor (MR) activation caused GEnGlx damage and albuminuria. In this study, we investigated whether MR antagonism could limit albuminuria in diabetes and studied the site of action. Streptozotocin-induced diabetic Wistar rats developed albuminuria, increased glomerular albumin permeability (P*s’_alb_*), and increased glomerular matrix metalloproteinase (MMP) activity with corresponding GEnGlx loss. MR antagonism prevented albuminuria progression, restored P*s’_alb_*, preserved GEnGlx, and reduced MMP activity. Enzymatic degradation of the GEnGlx negated the benefits of MR antagonism, confirming their dependence on GEnGlx integrity. Exposing human glomerular endothelial cells (GEnC) to diabetic conditions in vitro increased MMPs and caused glycocalyx damage. Amelioration of these effects confirmed a direct effect of MR antagonism on GEnC. To confirm relevance to human disease, we used a potentially novel confocal imaging method to show loss of GEnGlx in renal biopsy specimens from patients with diabetic nephropathy (DN). In addition, patients with DN randomized to receive an MR antagonist had reduced urinary MMP2 activity and albuminuria compared with placebo and baseline levels. Taken together, our work suggests that MR antagonists reduce MMP activity and thereby preserve GEnGlx, resulting in reduced glomerular permeability and albuminuria in diabetes.

## Graphical abstract

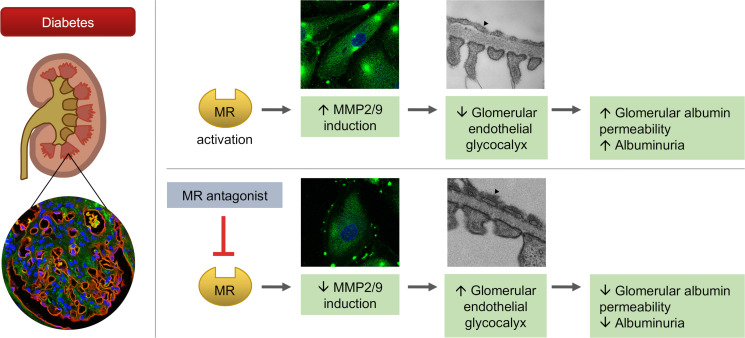

## Introduction

Glomerular diseases, including diabetic nephropathy (DN), are the most common cause of end-stage renal failure (1). Approximately 1 in 5 people with diabetes need treatment for DN during their lifetime (2). In 2018, more than 700,000 people in the United States were being treated for end-stage renal disease (ESRD), and diabetes accounted for 47% of all new ESRD cases (3). Renin-angiotensin-aldosterone system (RAAS) blockade with angiotensin-converting enzyme inhibitors (ACEi) or angiotensin-receptor blockers (ARB) reduce albuminuria and the risk of ESRD (4). However, because of aldosterone escape, use of an ACEi or ARB may not reduce aldosterone-mediated mineralocorticoid receptor (MR) stimulation (5, 6). In DN, the addition of an MR antagonist to ACEi or ARB therapy further reduces albuminuria, suggesting MR activation directly contributes to albuminuria (7–12). Most recently, the large Phase III FIDELIO-DKD trial reported that, in patients with chronic kidney disease (CKD) and type 2 diabetes, MR antagonism reduced the risk of CKD progression, albuminuria, and cardiovascular events (13). However, side effects, including hyperkalemia, limit the clinical use of MR antagonists (10, 12, 14–16). Thus, a better definition of the mechanisms of glomerular protection mediated by MR antagonists is needed to identify novel tissue-specific therapeutic targets.

MR is expressed in the vascular endothelium (17, 18) and is also expressed in glomerular endothelial cells (GEnC) (19) — highly specialized fenestrated vascular endothelial cells. Changes in the glomerular endothelium are increasingly recognized in DN and other glomerular diseases (20). The glomerulus is the filtering unit of the kidney. Its function is dependent on the multilayer structure of the glomerular filtration barrier (GFB) consisting of GEnC, glomerular basement membrane (GBM), and podocytes (21). The glomerular endothelial glycocalyx (GEnGlx) covers the luminal surface of the GEnC, filling the fenestrations and contributing to GFB function (22, 23). Our group and others have shown that GEnGlx specifically limits albumin permeability in vitro (24–26) and in vivo (27–30). The EnGlx is a hydrated poly-anionic gel composed principally of proteoglycan core proteins, glycosaminoglycan chains, and sialoglycoproteins (31). In healthy vascular physiology, the EnGlx has multiple roles, including regulating vascular permeability (32, 33), mediating shear stress mechanotransduction (34, 35), and attenuating immune cell–endothelium interactions (36, 37), with further roles under investigation (31, 38, 39). Disruption of the EnGlx occurs in multiple clinical conditions, including diabetes, sepsis, preeclampsia, and atherosclerosis (22, 31, 38, 40).

In humans, DN is characterized by albuminuria (22, 41). In early DN, no macroscopic GBM or podocyte changes are detectable, but systemic endothelial and Glx dysfunction in both type 1 (42) and type 2 (43) diabetes have been shown to occur. Others have previously shown that glycosaminoglycans are lost from the GFB in diabetes (44, 45), and we have confirmed GEnGlx loss in diabetic mice (46, 47) and rats (30). Together, these findings strongly implicate GEnGlx damage as a key initiator of albuminuria in DN (22, 23, 41).

EnGlx components are cleaved from the cell surface by sheddases, including matrix metalloproteinases (MMPs) (48, 49). We have recently defined a pathway whereby excess MR activation results in increased MMP2 and MMP9 activity and consequent GEnGlx dysfunction and albuminuria (19). Here, we sought to determine whether this pathological pathway contributes to GEnGlx damage in diabetes, hypothesizing that MR antagonism reduces MMP activity in diabetes, preserving the GEnGlx and limiting the development of albuminuria. Hence, we sought to determine whether MR antagonism, with spironolactone, could prevent the development of albuminuria in a DN rat model by preserving the GEnGlx to maintain the GFB. Furthermore, we examined GEnGlx damage and MR-mediated MMP inhibition in human DN.

## Results

### Development of albuminuria and increased glomerular permeability in early DN is ameliorated by MR antagonism.

**Figure 1:**
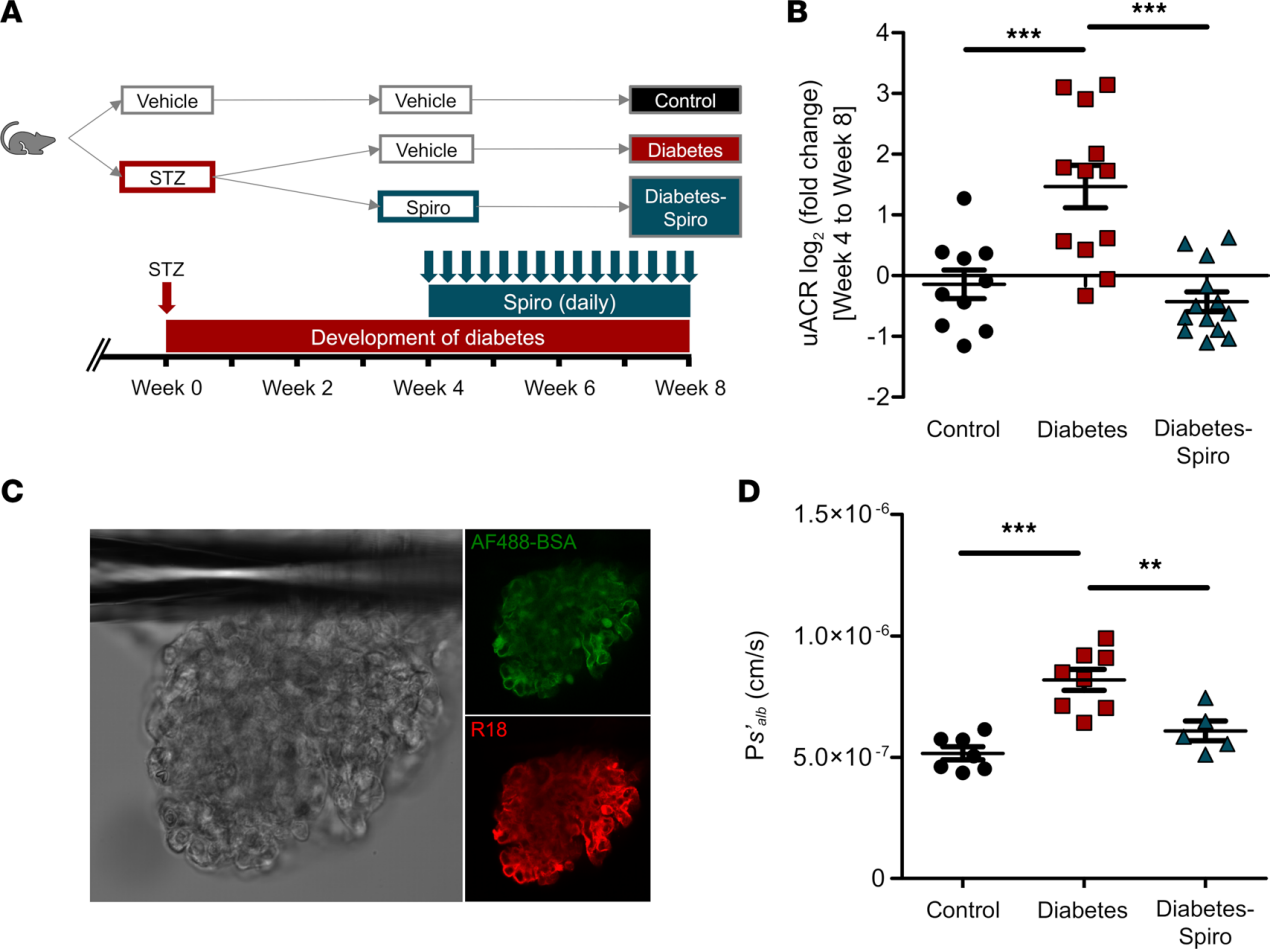
Development of albuminuria and increased glomerular permeability in early diabetic nephropathy is ameliorated by MR antagonism. (**A**) Schematic overview of STZ-induced diabetic model and spironolactone (spiro) treatment protocol for male Wistar rats. An injection of STZ was given at week 0. Four weeks after STZ injection, spiro (an MR inhibitor) was given for 28 days, and rats were culled at week 8 after STZ injection. Rats were randomized to receive STZ and spiro. (**B**) Treatment with spiro for 28 days reduced the fold change in urinary albumin/creatinine ratio (uACR) from initiation of treatment, week 4 to week 8 (control, *n* = 10; diabetes, *n* = 12; diabetes-spiro, *n* = 13). Data were log transformed and presented as log_2_ (fold change). (**C**) Representative images of an isolated glomerulus stained with R18 and Alexa Fluor 488–BSA (AF488-BSA). Magnification, 20***×***. (**D**) Glomerular albumin permeability (P*s’_alb_*) was measured at week 8 (control, *n* = 7 rats [32 glomeruli]; diabetes, *n* = 8 [36 glomeruli]; diabetes-spiro, *n* = 5 [21 glomeruli]). In **B** and **D**, 1-way ANOVA was used for statistical analysis, followed by Tukey’s multiple comparisons. Each dot, triangle, and square on the graph represents a rat. Data are expressed as mean ± SEM. **P < 0.01; ***P < 0.001.

After receiving streptozotocin (STZ), rats were hyperglycemic at week 3 (Supplemental Figure 1A; supplemental material available online with this article; https://doi.org/10.1172/jci.insight.154164DS1). STZ-induced diabetic rats gained significantly less weight than controls. Spironolactone treatment had no significant impact on body weight (Supplemental Figure 1B). Diabetic rats developed albuminuria by week 4 (Supplemental Figure 1C). Spironolactone significantly reduced albuminuria in diabetic rats compared with vehicle. The fold change in urinary albumin/creatinine ratio (uACR) in vehicle-treated diabetic rats significantly increased compared with controls and spironolactone-treated diabetic rats (Figure 1B). There was no significant difference in uACR fold change between spironolactone-treated diabetic rats and controls. Similarly, diabetic rats had a significant increase in fold change of total protein/creatinine ratio (Supplemental Figure 1D). Our glomerular permeability assay was used to directly measure the albumin permeability (P*s’_alb_*) of individually trapped glomeruli (30). In contrast to urine-based measurements, this ex vivo assay directly measures the GFB permeability to albumin in isolation, independently of hemodynamic factors and tubular albumin handling (Figure 1C). Increased glomerular capillary wall protein permeability was confirmed by an increase in P*s’_alb_* (Figure 1D). Spironolactone restored P*s’_alb_* to control values, significantly reducing P*s’_alb_* compared with vehicle-treated diabetic rats.

### Diabetes-induced GEnGlx damage in early DN is prevented by MR antagonism.

**Figure 2:**
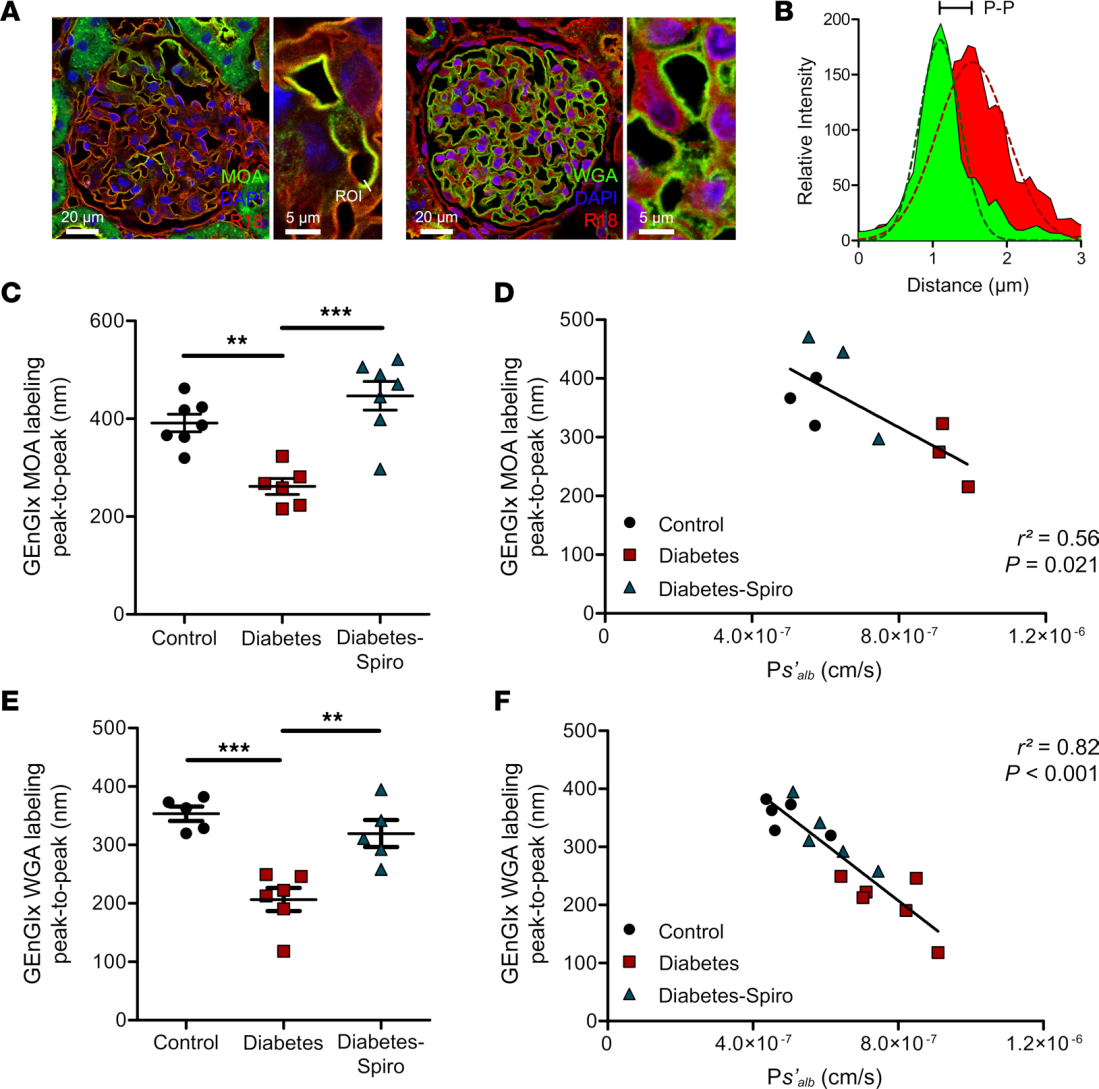
Fluorescence profile peak-to-peak measurements confirm that glomerular endothelial glycocalyx damage is prevented by MR antagonism and correlates strongly with glomerular albumin permeability. Rats were perfused with Ringer solution, and the left kidney was removed for lectin staining. (**A**) Representative images show glomerular capillaries labeled red (R18) and the luminal glomerular endothelial glycocalyx (GEnGlx) labeled green with *Marasmium oreades* agglutinin (MOA) or wheat germ agglutinin (WGA). Scale bars: 20 μm and 5 μm. ROI, region of interest for fluorescence profile peak-to-peak (P-P)measurement. (**B**) Representative relative intensity peaks of R18 (red) and MOA (green) profiles showing P-P assessment of the GEnGlx; Gaussian curves (dashed lines) were fit to the raw intensity data of each plot for P-P measurements. (**C** and **D**) Quantification at week 8 after STZ of GEnGlx MOA labeling P-P (control, *n* = 7; diabetes, *n* = 6; diabetes-spironolactone [diabetes-spiro], *n* = 7) and functional association with the rate of glomerular albumin leakage (P*s’_alb_*) (*n* = 9). (**E** and **F**) Quantification at week 8 after STZ of GEnGlx WGA labeling P-P (control, *n* = 5; diabetes, *n* = 6; diabetes-spiro, *n* = 5) and functional association with the rate of glomerular P*s’_alb_* (*n* = 16). In **C** and **E**, 1-way ANOVA was used for statistical analysis, followed by Tukey’s multiple comparisons. Each dot, triangle, and square on the graph represents a rat. Data are expressed as mean ± SEM. **P < 0.01; ***P < 0.001.

*Marasmium oreades* agglutinin (MOA) and wheat germ agglutinin (WGA) lectins both bound to the EnGlx on the luminal surface of the labeled GEnC membrane (Figure 2A). We analyzed the images generated using a fluorescence profile peak-to-peak measurement technique (19, 47, 50) to provide an index of GEnGlx thickness (Figure 2B). Manual peak-to-peak measurement of MOA labeling demonstrated a reduction in GEnGlx thickness in diabetic rats (Figure 2C). Spironolactone treatment in diabetic rats restored the GEnGlx thickness, with no significant differences compared with controls. P*s’_alb_* correlated inversely with the GEnGlx thickness measured by peak-to-peak analysis of MOA/R18 labeling (Figure 2D). To confirm the validity of our findings with MOA labeling, we applied a second lectin, WGA, for peak-to-peak measurements, and we developed an automated methodology. As with MOA, we found significant Glx damage in diabetic rats, with a decrease in GEnGlx thickness (Figure 2E). Spironolactone treatment significantly restored the GEnGlx thickness, with no significant differences compared with controls. Again, P*s’_alb_* changes correlated inversely, and strongly, with GEnGlx thickness measured from peak to peak of WGA/octadecyl rhodamine B chloride (WGA/R18) labeling (Figure 2F). Perfusion-fixed, Alcian blue–labeled, kidneys were used for transmission electron microscopy (TEM) of the glomerular capillary wall to validate peak-to-peak assessment of the Glx (Supplemental Figure 2A) and study GFB changes (Supplemental Figure 3A) (30, 46). Diabetic rats had decreased GEnGlx coverage and thickness, which were both restored by spironolactone treatment (Supplemental Figure 2, B and C). P*s’_alb_* changes were weakly associated with GEnGlx thickness measured by electron microscopy (EM) (Supplemental Figure 2D), suggesting that peak-to-peak assessment provides a superior measure of Glx structural and functional integrity. Mesangial matrix expansion was assessed using periodic acid–Schiff staining, with no significant changes in glomerular fibrosis in diabetic rats (Supplemental Figure 1, E and F). In addition, comprehensive TEM analysis confirmed that no other significant ultrastructural changes were visible (Supplemental Figure 3, B–H), further confirming that this model represents early DN.

### The effect of MR antagonism in preventing the diabetes-induced increase in glomerular permeability is dependent on the GEnGlx.

**Figure 3:**
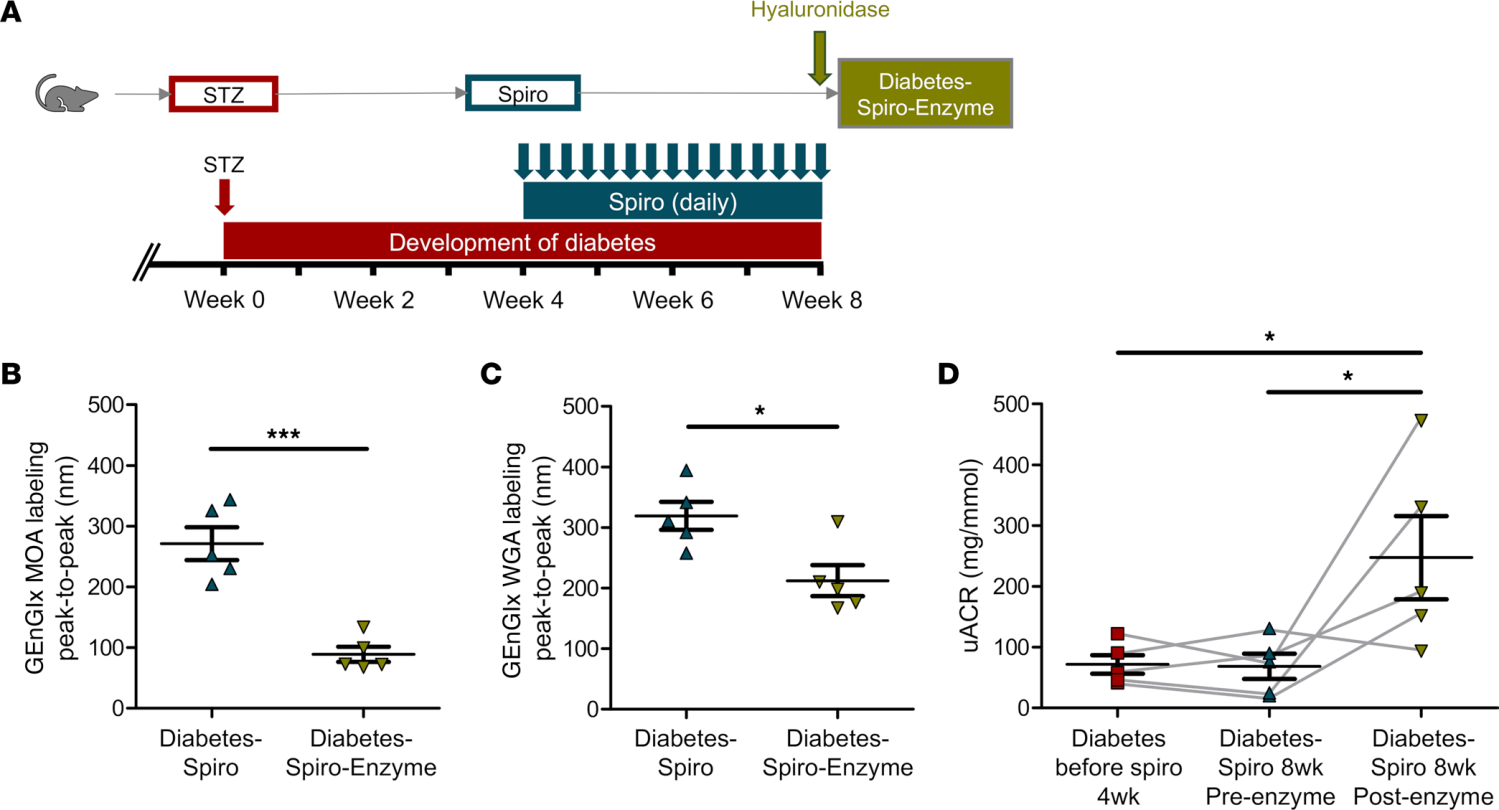
The effect of MR antagonism in preventing the diabetes-induced increase in glomerular permeability is dependent on the glomerular endothelial glycocalyx. (**A**) Schematic overview of enzymatic degradation of the glomerular endothelial glycocalyx (GEnGlx) with hyaluronidase on spironolactone-treated (spiro-treated) male Wistar rats. An injection of STZ was given at week 0. Four weeks after STZ injection, spiro (an MR inhibitor) was given for 28 days, and rats were given hyaluronidase (200 units) at week 8 after STZ via tail vein injection 1 hour before being culled for tissue collection. Rats were randomized to receive hyaluronidase. (**B** and **C**) Quantification at week 8 after STZ of GEnGlx WGA labeling peak-to-peak (diabetes-spiro, *n* = 5; diabetes-spiro-enzyme, *n* = 5) and GEnGlx MOA labeling peak-to-peak (diabetes-spiro, *n* = 5; diabetes-spiro-enzyme, *n* = 5) confirmed enzyme degradation of GEnGlx. In **B** and **C**, unpaired *t* test was used for statistical analysis. (**D**) Albuminuria levels returned to those expected in vehicle treated diabetic rats. Urinary albumin/creatinine ratio (uACR) was determined from the same rats (*n* = 5) at week 4 after STZ (diabetes before spiro 4wk), week 8 after treatment with spiro (diabetes-spiro 8wk preenzyme), and week 8 after hyaluronidase (diabetes-spiro 8wk post-enzyme). The connecting line (gray) represents the same rat for each data point. Repeated-measures 1-way ANOVA was used for statistical analysis, followed by Tukey’s multiple comparisons. Each triangle or square on the graph represents a rat. Data are expressed as mean ± SEM. *P < 0.05; ***P < 0.001.

Hyaluronidase, a Glx-degrading enzyme, was infused in a subgroup of spironolactone-treated diabetic rats to remove the EnGlx to confirm the importance of GEnGlx preservation in this model (Figure 3A). Automated peak-to-peak measurement of both MOA and WGA labeling confirmed significant reductions in GEnGlx thickness following enzyme infusion (Figure 3, B and C). Enzymatic degradation of the GEnGlx significantly increased the uACR and returned albuminuria to diabetes-induced levels (Figure 3D). TEM image analysis confirmed that hyaluronidase caused significant GEnGlx loss (Supplemental Figure 4, A–C). No significant effect on either podocyte Glx (pGlx) or other filtration barrier components were detectable (Supplemental Figure 4, D–J), confirming that hyaluronidase activity remained focused on the luminal EnGlx.

### Increased MMP activity in early DN is ameliorated by MR antagonism.

**Figure 4:**
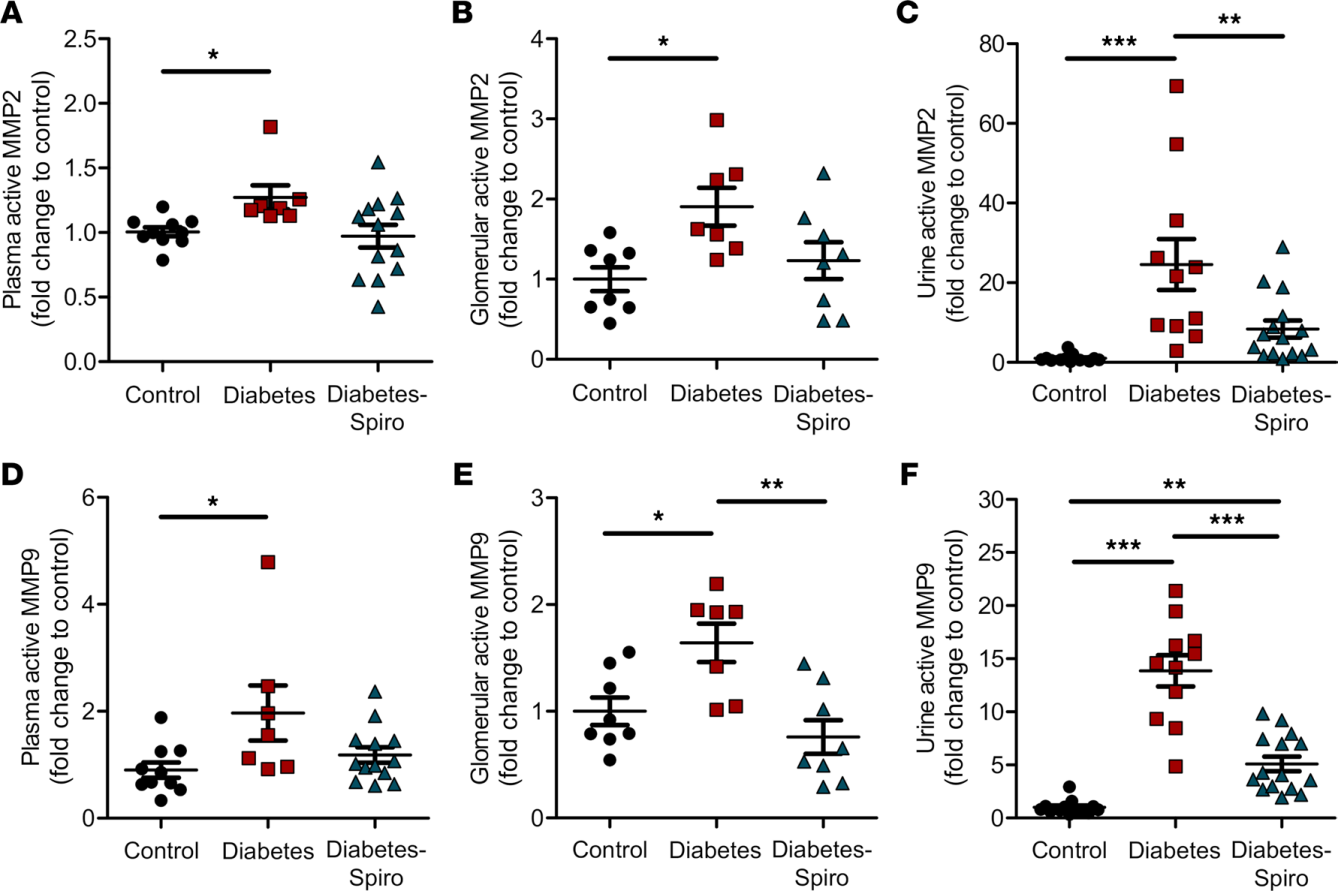
Increased matrix metalloproteinase activity in early diabetic nephropathy is ameliorated by MR antagonism. (**A** and **D**) Matrix metalloproteinase (MMP) activities were measured for the sheddases MMP2 and MMP9. Systemic circulation of plasma active MMP2 (**A**) and plasma active MMP9 (**D**) (control, *n* = 10; diabetes, *n* = 7; diabetes-spironolactone [diabetes-spiro], *n* = 13) were determined. (**B** and **E**) Localized activity of glomerular active MMP2 (**B**) and glomerular active MMP9 (**E**) (control, *n* = 8; diabetes, *n* = 7; diabetes-spiro, *n* = 8) were determined from isolated glomeruli and normalized to glomerular total protein. (**C** and **F**) Urine active MMP2 (**C**) and urine active MMP9 (**F**) (control, *n* = 13; diabetes, *n* =11; diabetes-spiro, *n* = 15) were determined and normalized to urine creatinine. Each dot, triangle, and square represents a rat. The fold change relative to control was calculated to enable pooling of results from different experiments. One-way ANOVA was used for statistical analysis, followed by Tukey’s multiple comparisons. Data are expressed as mean ± SEM. *P < 0.05; **P < 0.01; ***P < 0.001.

We have previously identified MMP2 and MMP9 as key Glx sheddases (19, 47, 49). Plasma, glomerular, and urine active MMP2 were significantly increased in diabetic rats, compared with controls (Figure 4, A–C). Similarly, plasma, glomerular, and urine active MMP9 were significantly increased in diabetic rats compared with controls (Figure 4, D–F). Therapeutic treatment with spironolactone significantly reduced glomerular active MMP9, with no significant differences compared with controls (Figure 4E). In addition, spironolactone significantly attenuated the diabetes-induced increase in both urine MMP2 and MMP9 activities (Figure 4, C and F), confirming that MR antagonism reduced the activity of both MMPs in vivo.

### Exposing human GEnC to diabetic conditions resulted in MMP upregulation and GEnGlx damage that were both ameliorated by MR antagonism.

**Figure 5:**
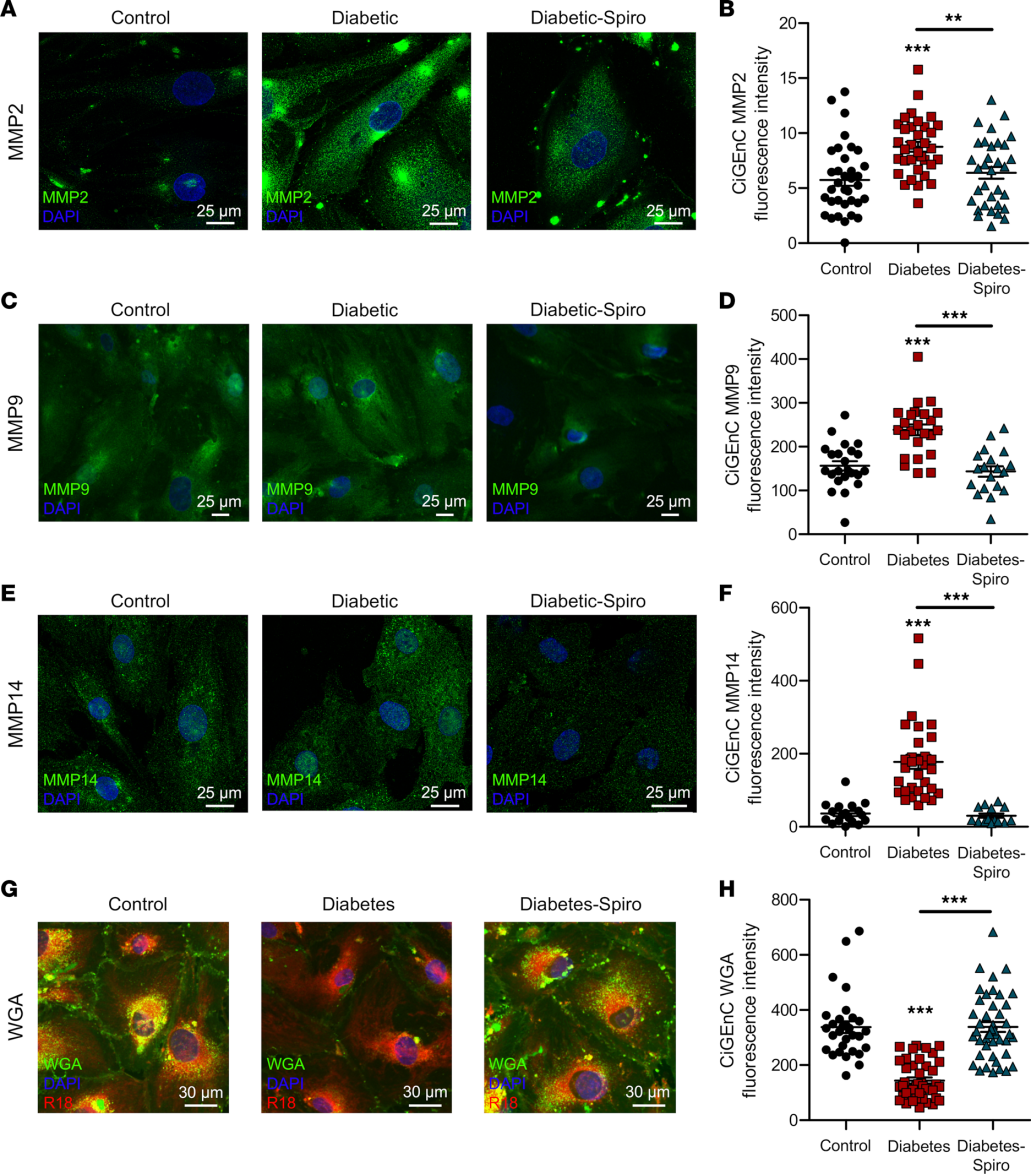
Exposing human GEnC to diabetic conditions resulted in MMP upregulation and GEnGlx damage, effects ameliorated by MR antagonism. Human conditionally immortalized glomerular endothelial cells (CiGEnC) were maintained in the presence of glucose, insulin, TNF-α, and IL-6 to mimic a diabetic environment. (**A**, **C**, **E**, and **G**) Representative images of CiGEnC stained with MMP2, MMP9, MMP14, or WGA lectin (an endothelial glycocalyx label) shown for control, diabetes, and diabetes-spironolactone (diabetes-spiro) samples. DAPI, nuclear label; R18, endothelial membrane label. Scale bars: 25 μm. (**B**, **D**, **F**, and **H**) Fluorescence intensity was quantified in CiGEnC for MMP2 (control, *n* = 35; diabetes, *n* = 34; diabetes-spiro, *n* = 33), MMP9 (control, *n* = 23; diabetes, *n* = 24; diabetes-spiro, *n* = 19), MMP14 (control, *n* = 18; diabetes, *n* = 31; diabetes-spiro, *n* = 14), and WGA lectin (control, *n* = 30; diabetes, *n* = 41; diabetes-spiro, *n* = 43). In **A** and **B**, 1-way ANOVA was used for statistical analysis, followed by Tukey’s multiple comparisons. In **C** and **D**, Kruskal-Wallis test was used for statistical analysis. Data are expressed as mean ± SEM. **P < 0.01; ***P < 0.001.

Secreted gelatinases MMP2 and MMP9 can be activated by cell membrane–bound membrane type 1 MMP, also known as MMP14 (51–53). We therefore investigated if localized endothelial production and activation could contribute to GEnCGlx degradation in diabetes. To mimic the diabetic milieu in vitro, conditionally immortalized (Ci) human GEnC were exposed to increased concentrations of glucose, insulin, TNF-α, and IL-6 (54). MMP2, MMP9, and MMP14 expression significantly increased under diabetic conditions (Figure 5, A–F). Spironolactone prevented these increases. WGA lectin glycocalyx staining (Supplemental Figure 5, A and B) was reduced following exposure to diabetic conditions, and spironolactone limited the reduction in WGA lectin glycocalyx staining, suggesting glycocalyx preservation (Figure 5, G and H). In addition, *MMP2*, *MMP9*, and *MMP14* gene expression was significantly increased in GEnC exposed to diabetic conditions (Supplemental Figure 5, C–E). Spironolactone reduced this diabetes-induced increase in *MMP* mRNA, although this was only significant with *MMP14*. These effects happen in isolation from other cell types, demonstrating a direct targeted effect of spironolactone on GEnC.

### The GEnGlx is damaged in human DN.

**Figure 6:**
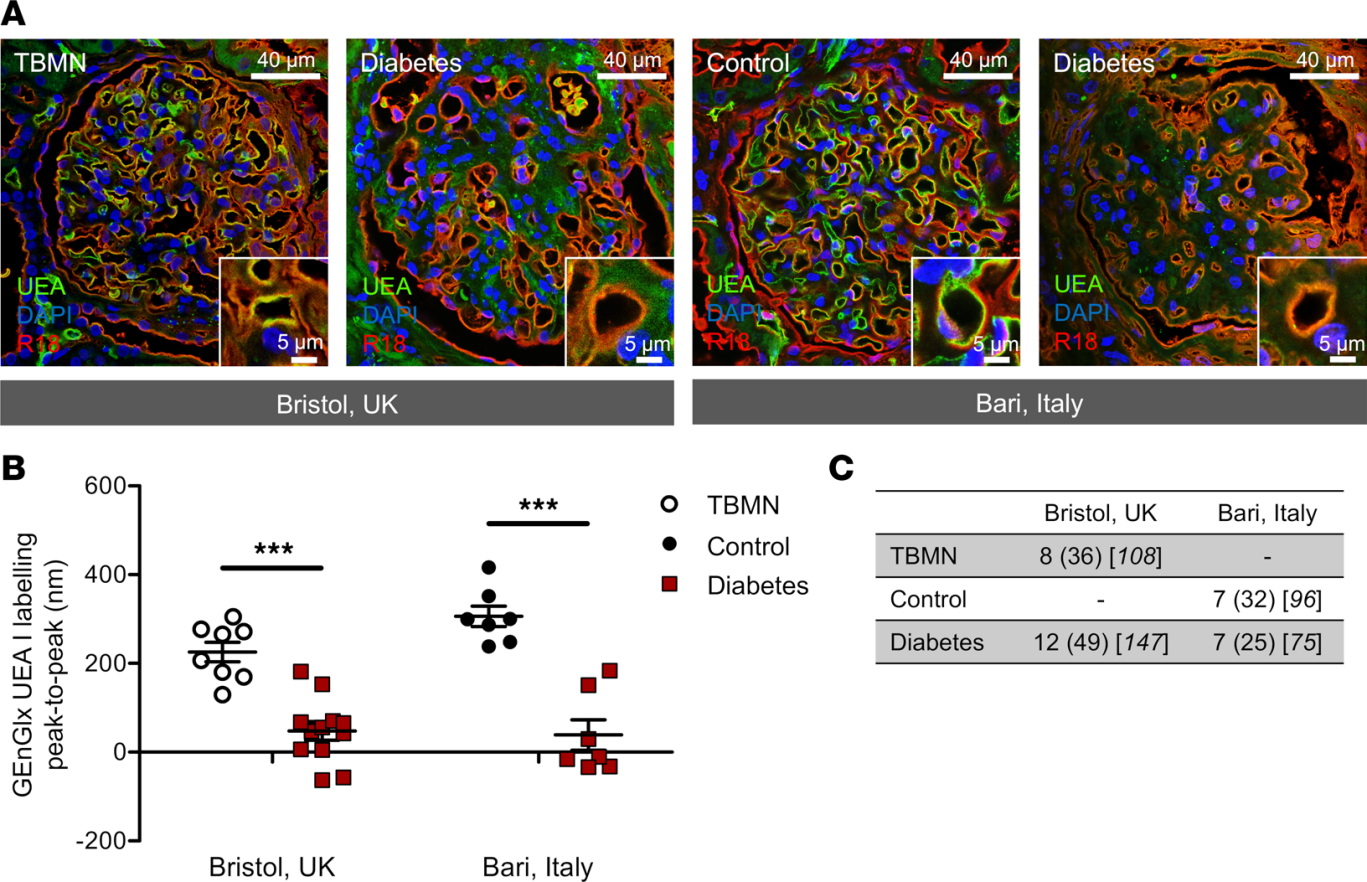
The glomerular endothelial glycocalyx is damaged in human diabetic nephropathy. (**A**) Representative images show glomerular capillaries labeled red (R18) and the luminal glomerular endothelial glycocalyx (GEnGlx) labeled green with *Ulex europaeus* agglutinin I (UEA-I) in renal biopsies from thin basement membrane nephropathy (TBMN) and diabetic nephropathy (DN) patients from Bristol, United Kingdom, and from histologically normal controls and patients with DN from Bari, Italy. Scale bar: 40 μm and 5 μm. (**B**) Quantification of GEnGlx UEA labeling peak-to-peak (Bristol: TBMN, *n* = 8; diabetes, *n* = 12; Bari: control, *n* = 7; diabetes, *n* = 7) confirms that GEnGlx damage may contribute to the disease phenotype seen in human DN. Unpaired *t* test was used for statistical analysis. (**C**) The number of samples, glomeruli, and capillaries used to analyze each group. Each dot or square on the graph represents a patient. Data are expressed as mean ± SEM. ***P <0.001.

Human renal biopsies were obtained from two centers (Bristol, United Kingdom; Bari, Italy) (Table 1). *Ulex europaeus* agglutinin I (UEA-I) lectin has been established as an excellent marker for human endothelial cells (55, 56) and binds specifically to the EnGlx on the luminal surface of the GEnC (Figure 6A). Representative images highlight the reduction in UEA-I labeling in renal biopsies from patients with DN when compared with thin basement membrane nephropathy (TBMN) controls or histologically normal controls. Following extensive validation in rodents, our peak-to-peak measurement technique has allowed us to confirm that GEnGlx damage occurs in human DN and is likely to contribute to the disease phenotype. Peak-to-peak measurement of UEA-I labeling, with our blinded automated methodology, demonstrated a significant reduction in GEnGlx thickness in patients with DN (Figure 6, B and C). This was observed in renal biopsies from both the Bristol and Bari cohorts, when compared with TBMN controls or histologically normal controls respectively.

### MR antagonism in human diabetes reduces urinary MMP activity.

**Figure 7:**
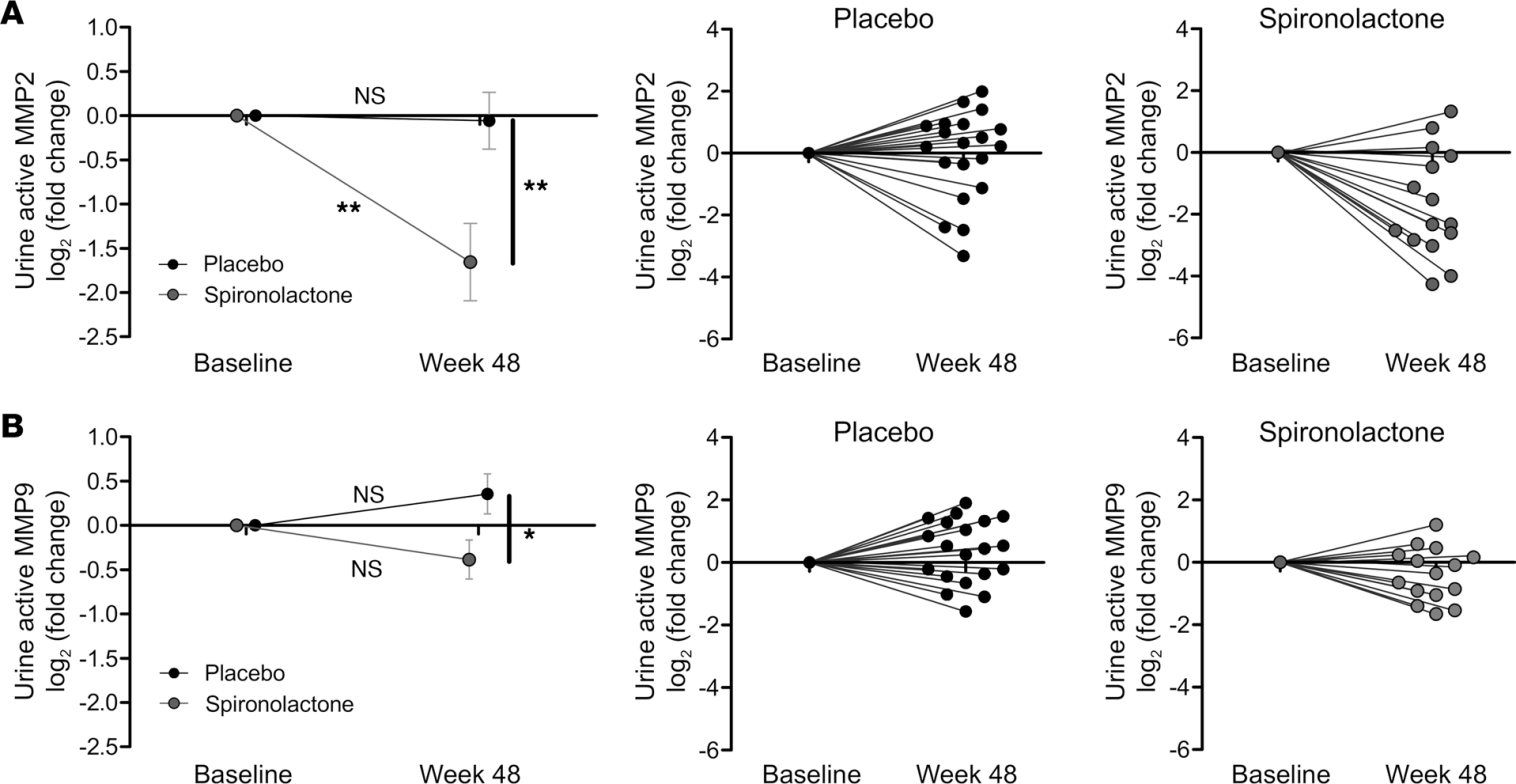
Matrix metalloproteinase activity in human diabetes is reduced by MR antagonism. Matrix metalloproteinase (MMP) activities were measured for the sheddases MMP2 and MMP9. (**A** and **B**) Urine active MMP2 and urine active MMP9 were determined and normalized to urine creatinine (placebo, *n* = 20; spironolactone, *n* = 15). Data were log transformed and presented as log_2_ (fold change). Mean fold change in placebo and spironolactone groups were presented for urine active MMP2 and urine active MMP9. Normalized changes for each individual taking placebo/spironolactone are also displayed to illustrate the variability in individuals’ progression with time/response. Paired *t* test was used for statistical analysis between baseline and week-48 data. Data are expressed as mean ± SEM. *P < 0.05; **P < 0.01. Individual data for urine active MMP fold change from baseline to week 48 were presented for placebo and spironolactone groups. Each dot represents an individual patient.

Previously, Mehdi et al. confirmed that the addition of spironolactone to a regimen including maximal ACE inhibition improved renoprotection in DN (57). In this study, the significant reduction in albuminuria (compared with placebo and baseline) occurred despite no significant effect on ambulatory or clinic blood pressure (BP). Urine samples obtained from this clinical trial (Table 2) confirmed that, after 48 weeks of treatment, both urine active MMP2 and MMP9 in spironolactone-treated patients were significantly reduced (relative to placebo-treated patients) (Figure 7, A and B). Urine active MMP2 level decreased significantly from baseline in the spironolactone group but not in the placebo group (Figure 7A). The response to intervention for each individual subject demonstrated that 12 of 15 participants randomized to spironolactone experienced a decrease in the urinary MMP2 activity (Figure 7A). The effects on MMP9 were more modest, as previously reported, in human disease (58).

## Discussion

Using a rat model of diabetes, we have demonstrated that upregulation of MMPs, GEnGlx damage, and glomerular dysfunction develop rapidly before other visible markers of DN. MR antagonism preserved the GEnGlx and restored GFB function. In vitro, using human GEnC exposed to a diabetic environment, we also observed upregulation of MMPs and glycocalyx damage, effects ameliorated by MR antagonism. In addition, we confirmed that GEnGlx damage occurs in human DN and may contribute to the disease phenotype. Finally, we have demonstrated that MR antagonism in human DN reduced MMP activity, with associated reductions in albuminuria. Together, these data suggest that alternative approaches to limit MMP activity and GEnGlx damage may reproduce the effect of MR inhibition while limiting side effects.

STZ-induced diabetic rats provide a good model of early changes in DN (30, 59, 60). Experiments using pancreatic islet transplantation confirm that albuminuria is due to DN, not STZ toxicity (61). Our diabetic rats progressively developed albuminuria; however, we found that urinary albumin excretion is not a sensitive measure of glomerular permeability, likely due to variable tubular albumin reabsorption and local hemodynamic alterations. Our glomerular P*s’_alb_* assay directly measures the albumin permeability of capillary loops within isolated glomeruli, removing the effect of tubular albumin reuptake and hemodynamic factors (including changes in systemic BP) (30). We observed an increase in glomerular P*s’_alb_* in early DN, and both P*s’_alb_* and albuminuria were limited by MR antagonism, confirming a direct action on the GFB.

The GEnGlx plays an important role in GFB function, limiting albumin permeability (22, 23, 28). TEM is often used to directly visualize and measure the EnGlx (62–64); however, damage to the EnGlx during tissue preparation can introduce variability (62, 63). Sample fixation also affects quantification of EnGlx, with perfusion-fixation rather than immersion-fixation of tissue preferred (65). However, variable tissue perfusion in disease potentially confounds this technique, with a subjective increase in renal perfusion variability noted in diabetic animals. Furthermore, perfusion-fixation techniques are not applicable in humans. TEM analysis showed reduced GEnGlx coverage in diabetic rats, restored by inhibiting MR. However, TEM-derived GEnGlx measures showed only a weak correlation with P*s’_alb_*. In mice, MOA lectin is known to bind to specific carbohydrate sequences present in the GEnGlx (47, 66, 67), and here, we confirmed that it bound to the Glx on the luminal surface of the R18-labeled GEnC in rats. WGA lectin is also known to bind to the EnGlx in rats (28, 50, 68), and with MOA, lectin was used to study the GEnGlx. Our peak-to-peak technique has previously been used, in vivo (19, 50) and on fixed kidney tissue (47), to provide an index of Glx thickness. Using both MOA and WGA lectin labeling, we have demonstrated that diabetes-induced reductions in GEnGlx thickness are restored by MR antagonism. In addition, GEnGlx thickness measured by peak-to-peak image analysis correlated with P*s’_alb_* more strongly than TEM-based measurements. We find that peak-to-peak measurements (using optimized lectin/R18 labeling) are consistently thicker than measures using TEM and are closer to estimates made using atomic force microscopy (69–72) and solute exclusion (73–75). This is likely due to variable perfusion-fixation and EnGlx collapse during the dehydration process required for TEM imaging (62, 63, 76). These findings further validate our peak-to-peak image analysis technique as a cheap, reliable, and robust alternative measure of EnGlx changes. Other glomerular ultrastructural features were not affected in this early disease model; for instance, STZ-induced diabetic rats did not develop GBM thickening or podocyte effacement by 8 weeks. In addition, no glomerular fibrosis was observed. These data confirm our previous observations that isolated disruption of GEnGlx in early DN is associated with albuminuria and increased P*s’_alb_* (30).

Hyaluronan is a key Glx component that is shed in diabetes (77). Using hyaluronidase to enzymatically degrade the GEnGlx in spironolactone-treated diabetic rats, we demonstrated that stripping the restored Glx increased albuminuria back to diabetes-induced levels, confirming the importance of GEnGlx preservation in this model. Previously in mice, using a combination of hyaluronidase and chondroitinase, we have shown that enzymatic GEnGlx depletion resulted in a significant increase in endothelial permeability (30, 78). Here, we used hyaluronidase in isolation hypothesizing that the short half-life in circulation (3.2 minutes in rats) (79) and molecular weight (61 kDa) would limit its effects to the EnGlx. TEM confirmed that hyaluronidase reduced the GEnGlx, without affecting pGlx or other glomerular components, confirming that focused, isolated glycocalyx injury occurs following acute hyaluronidase infusion, as seen following chronic hyaluronidase infusion (80).

MR-induced MMPs are likely to contribute to Glx damage in DN. In mice, we have demonstrated increased MMP activity induced by excess aldosterone (19) and diabetes (47) and have shown that inhibiting MMPs directly afforded significant GEnGlx protection (19, 47). Here we have shown increased plasma, glomerular, and urine active MMP2 and MMP9 in diabetic rats and have shown successful inhibition of this effect using the MR antagonist spironolactone, a drug already approved for clinical use. These findings are consistent with our previously published work using vascular endothelial growth factor A_165_b, angiopoietin-1, and an MMP2/9 inhibitor where, in each case, GEnGlx restoration successfully reduced glomerular permeability in early DN (30, 46, 47).

MR is widely expressed by vascular endothelial cells, including GEnC, (17–19), but it is also expressed in a variety of other cell types within the kidney (81–86) and blood (87–90). These various targets may contribute to the beneficial effects of the MR antagonism in DN. However, we have shown previously that GEnC upregulate *MMP2* and *MMP9* mRNA in response to diabetic conditions or aldosterone excess in vitro, with associated increases in MMP activity suggesting that a direct effect is likely (19, 47). In this study, we observed an increase in glomerular P*s’_alb_* following diabetes onset that was reduced by MR antagonism measured using an ex vivo assay (30) independent of hemodynamic factors and tubular albumin reuptake. This confirmed a direct action of MR antagonism on the GFB. When studied by EM and light microscopy, the only detectable changes in this model were focused within the GEnGlx. Furthermore, utilizing human CiGEnC, we have shown increased MMP2, MMP9, and MMP14 protein expression in GEnC exposed to chronic diabetic conditions, with an associated reduction in Glx. MR antagonism with spironolactone successfully inhibited these effects, suggesting a direct targeted effect of spironolactone on GEnC. To further investigate the key sites of MR antagonism, future work is planned utilizing cell type–specific deletion of MR in DN models.

In humans, the absence of GBM and podocyte changes in early disease, along with systemic endothelial and Glx dysfunction seen in both type 1 (42) and type 2 (43) diabetes, strongly implicate GEnGlx damage as a key early event in DN development (22, 23, 41). Here we have confirmed that GEnGlx damage occurs in human DN and may contribute to the disease phenotype. Great care has been taken to ensure that all samples were collected and processed identically. This ensured the Glx alterations seen between healthy, TBMN, and DN biopsies were the result of the primary pathology, not the collection technique. Confirming Glx damage in human DN highlights the importance of identifying therapeutic targets capable of limiting EnGlx degradation in disease. Such therapies could be of great benefit in both glomerular and systemic vascular disease (91–93). Previously, the addition of spironolactone to a regimen including maximal ACE inhibition has been shown to afford greater renoprotection in human DN (57). Compared with placebo, the uACR significantly decreased following spironolactone. Importantly in this study, BP treatment response was no different between placebo and spironolactone groups, suggesting a mechanism of action beyond BP control (57). However, the serum potassium level was significantly higher following the addition of spironolactone (57), and hyperkalemia continues to limit the clinical use of MR antagonists (10, 12–16). Most recently, the FIDELIO-DKD trial reported that MR antagonism, with finerenone, lowered the risk of CKD progression, reduced albuminuria, and reduced cardiovascular events in patients with CKD and type 2 diabetes, further highlighting the utility of MR antagonists in clinical practice (13). Previously, studies in patients with type 1 and type 2 diabetes have found increased plasma MMP2 levels (58, 94, 95) and elevated activities of urinary MMP2 and MMP9 (96–98). Spironolactone treatment has been shown to reduce MMP2 and MMP9 levels in patients at risk of heart failure (99–101). Using urine obtained from the Mehdi et al. clinical trial (57), we have shown that the addition of MR antagonism to maximal ACE inhibition in patients with DN reduced urine active MMP2 and MMP9 compared with placebo, with an associated reduction in albuminuria. This highlights that common mechanisms of Glx damage are likely in both human DN and rodent models of disease.

In summary, we have shown that MR antagonism prevented damage to the GEnGlx and normalized glomerular albumin permeability in early DN. By applying potentially novel, validated techniques, we have demonstrated that GEnGlx damage may contribute to the disease phenotype in human DN. In addition, MR antagonism in DN reduced MMP activity, highlighting a mechanism of Glx protection. Alternative approaches to block MR-induced GEnGlx dysfunction, to reproduce the benefit of MR antagonists in DN without the adverse effects, warrant further investigation.

## Methods

### Animals.

Male Wistar rats (150–200 g, Charles River Laboratories) were maintained by the Animal Services Unit, University of Bristol. Animals were housed in a conventional facility with a controlled environment (21°C–24°C and 12:12 hour light/dark cycle). Timelines of the experimental protocols used are included (Figure 1A and Figure 3A).

### Type 1 diabetic model.

For induction of diabetes, randomized rats were injected i.p. with 50 mg/kg STZ (S0130; Sigma-Aldrich) by adding the appropriate volume of the drug at 25 mg/mL in 10 mM sodium citrate (pH 4.5). Comparisons were made with vehicle-treated rats (10 mM sodium citrate [pH 4.5]). Glycemia, by tail-tip blood droplet analysis using a glucometer (Accu-Chek Aviva; Roche), was measured 3 weeks after STZ, and rats with glycemia ≥ 15 mmol/L were considered diabetic and included in the study.

To confirm whether MR antagonism could preserve the GEnGlx and limit the development of DN, from 4 weeks after STZ administration, randomized diabetic rats were given daily s.c. injections of spironolactone (S3378; Sigma-Aldrich) at 50 mg/kg made up in corn oil (C8267; Sigma-Aldrich) for 28 days, and comparisons were made with vehicle-treated diabetic rats (corn oil). This spironolactone dose has previously been shown to have no effect on BP (102–105).

To determine the importance of GEnGlx preservation in this model, enzymatic degradation of the GEnGlx with hyaluronidase was used. Briefly, randomized diabetic rats treated with spironolactone were given hyaluronidase (H3506; Sigma-Aldrich), 200 units in 1 mL, via tail vein injection 1 hour before being culled for tissue collection.

Body weight was monitored regularly after STZ, and rats were placed on hydrophobic sand to collect urine. Urinary albumin was quantified with a rat albumin ELISA (E111-125, Bethyl Laboratories), and creatinine was measured using an enzymatic spectrophotometric assay (Konelab T-Series 981845; Thermo Fisher Scientific). uACR and urinary protein/creatinine ratio (uPCR) were calculated as previously described (46). Rats were culled at 8 weeks after STZ injection for tissue collection.

### Tissue collection.

For tissue collection, rats were anesthetized with isoflurane in 1 L/min oxygen. A midline laparotomy was performed, and the abdominal aorta was cannulated with PE-10 tubing (427400; Becton Dickinson) to flush both kidneys with Ringer solution (NaCl, 132 mM; KCl, 4.6 mM; MgSO_4_-7H_2_O, 1.27 mM; CaCl_2_-2H_2_O, 2 mM; NaHCO_3_, 25 mM; D(+)glucose, 5.5 mM; HEPES acid, 3.07 mM; HEPES sodium salt, 1.9 mM [pH 7.40]). The left kidney was then removed for lectin staining (1/4 of the kidney, 4% PFA fixed) and glomerular permeability assay (3/4 of the same kidney, sieved for glomeruli) (30). The right kidney was subsequently perfusion fixed with a solution containing 2.5% glutaraldehyde, 0.1M cacodylate, and 1% Alcian blue for TEM to quantify GEnGlx thickness and coverage (30, 46).

### Glomerular albumin permeability (Ps’_alb_) assay.

The glomerular P*s’_alb_* assay was carried out as previously described (30). Briefly, Ringer-perfused kidney was sieved in 4% BSA in Ringer solution. Isolated glomeruli were incubated in 36.5 μg/mL octadecyl rhodamine B chloride (R18) (O246; Thermo Fisher Scientific) for 15 minutes and were then washed in 4% Ringer BSA to remove unbound R18, followed by 15 minutes’ incubation in 30 μg/mL Alexa Fluor 488–BSA (A13100; Thermo Fisher Scientific). An individual glomerulus was trapped on a custom-made petri dish, and the perfusate was switched from 30 μg/mL labeled 488-BSA to 30 μg/mL unlabeled BSA. A Nikon Ti-E inverted confocal microscope (Nikon Instruments Inc.) was used to capture the fluorescence intensity. The rate of decline in fluorescence intensity within the loop of the capillaries for the first minute was used to calculate P*s’_alb_* as previously described (30). Observers were blinded to sample identity.

### Lectin staining.

MOA, WGA, and UEA-I lectins have binding specificities for carbohydrate sequences present in the glycocalyx. MOA lectin binds to nonreducing terminal galactose-α-1,3-galactose-carbohydrates, WGA lectin binds to the sialyloligosaccharides, N-Acetylglucosamine and N-Acetylneuraminic acid, and UEA-I binds to α-L-fucose. Lectins were labeled with biotin or FlTC: biotinylated MOA (Z8-BA-9001-1, TCS Biosciences, 2 mg/mL; 1:100); FITC-WGA (GTX01502; GeneTex; 5 mg/mL; 1:500); and biotinylated UEA-I (GTX01511; GeneTex; 2 mg/mL; 1:200):

Paraffin-embedded kidney sections (5 μm) were dewaxed in Histo-Clear II (National Diagnostics) followed by rehydration in graded ethanol and a wash in PBS. All sections were incubated in blocking buffer (1% BSA in PBS containing 0.1% Tween) for 30 minutes. For biotinylated lectins, this was followed by endogenous biotin blocking using a streptavidin/biotin blocking kit (SP-2002; Vector Laboratories). After 2 washes, the sections were incubated with the biotinylated lectin (pH 6.8) overnight at 4°C. Buffer only was used as a negative control. After 4 washes, the sections were incubated with streptavidin–Alexa Fluor 488 (1:500, S32354; Thermo Fisher Scientific) (pH 6.8) for 1 hour at room temperature. For FITC-labeled lectins, the sections were incubated overnight at 4°C (pH 6.8). Then, for all sections, the nuclei were counterstained with 4′,6-diamidino-2-phenylindole (D1306; Thermo Fisher Scientific) and the cell membrane was labeled with R18 (1:1,000, O246; Thermo Fisher Scientific) for 10 minutes at room temperature. After a 2-minute wash in PBS, the coverslips were mounted in Vectashield mounting medium (H-1000; Vector Laboratories) and examined using either an AF600 LX wide-field fluorescence microscope (Leica Microsystems) or a Leica SP5-II confocal laser scanning microscope attached to a Leica DMI 6000 inverted epifluorescence microscope.

### Fluorescent profile confocal peak-to-peak analysis.

Initially, the fluorescence profile peak-to-peak assessment was carried out, blinded using a manual methodology, as previously described (19, 50). A perpendicular profile line (ROI in Figure 2A) was drawn from the inside to the outside of the capillary loop crossing the lectin-labeled Glx first, followed by the R18 labeled endothelial membrane. Fluorescence intensity profiles were then generated for the lectin-labeled components of the EnGlx and endothelial cell label. The distance between the peak signals from the lectin-488 and the R18 labels (peak-to-peak) is an index of Glx thickness (Figure 2B). The mean was determined from an average of 3 lines per capillary loop, 3 loops per glomerulus, and 3 glomeruli per rat. Our peak-to-peak measurement technique was subsequently updated to use a blinded automated methodology. This increased the number of measurements taken from each capillary loop and reduced the time taken to analyze each glomerulus. Briefly, we developed an ImageJ (NIH) macro to take multiple measurements in a preselected capillary loop and generate fluorescence intensity profiles for the lectin components of the GEnGlx and endothelial cell label. Gaussian curves were applied to the raw intensity data of each plot for peak-to-peak measurements (dashed lines in Figure 2B). The mean was subsequently determined from 200 lines per capillary loop, 3 loops per glomerulus, and 4–6 glomeruli per rat. Data were excluded with a SD > 7.5 and/or a signal-to-noise ratio < 15.

### PAS staining.

Rat paraffin-embedded kidney sections from perfused kidneys were stained using a periodic acid–Schiff (PAS) kit according to the manufacturer’s instructions. Images were quantified with ImageJ using the Haematoxylin PAS color deconvolution plug-in. Corrected total cell intensity values were calculated as follows: integrated density (area of glomerulus × background mean gray value). Ten glomeruli were analyzed per rat.

### TEM.

Perfusion-fixed right kidneys were used for TEM, as described previously, to measure GEnC, GBM, and podocyte parameters to identify effects on GFB ultrastructure that could explain changes in glomerular permeability (30, 46). Electron micrographs were taken using a Technai 12 electron microscope (FEI), and image analysis was carried out using established protocols in 3–4 capillary loops per glomerulus and 2–3 glomeruli per animal (30, 46). Briefly, ImageJ (NIH) software (SciJava software ecosystem) was used to overlay a grid onto the electron micrograph. The anatomical distance from the luminal phospholipid at sequential grid intersections to the farthest point of the glycocalyx was measured as glycocalyx thickness. A glycocalyx thickness ≤ 10 nm was considered uncovered, and this was expressed as a percentage of total measurements taken on a grid section. GBM width, podocyte foot process width, and slit diaphragm width were also measured. Fenestration density and podocyte foot process density were measured by counting the number of each and dividing by the length of GBM used for analysis. Observers were blinded to sample identity.

### MMP activity.

MMP2 activity was studied using the MMP2 Biotrack activity assay (RPN2631; GE Healthcare) and the SensoLyte Plus 520 MMP2 assay (AS-72224; AnaSpec). MMP9 activity was studied using the SensoLyte Plus 520 MMP9 assay (AS-72017; AnaSpec). The manufacturer’s instructions were followed in full. Urinary active MMP2 and MMP9 were normalized to creatinine. Glomerular active MMP2 and MMP9 were normalized to total protein using the Pierce BCA protein assay (23225; Thermo Fisher Scientific). The fold change relative to control was calculated to enable pooling of results from different experiments.

### Cell culture.

Human CiGEnC (106) were maintained in EGM2 media (Lonza) supplemented with 10% FBS and EGM2-MV bullet kit (Lonza) in the absence of supplied gentamicin. Cells were differentiated at 37°C for 5 days (80% confluence) before entering the study and were free of mycoplasma infection. To mimic a diabetic environment in vitro, CiGEnC were maintained in the presence of 100 nmol/L insulin (Tocris), 25 mmol/L glucose (MilliporeSigma), 1 ng/mL TNF-α, and 1 ng/mL IL-6 (both from R&D Systems) (54). To study alterations in gene expression, CiGEnC were pretreated for 2 hours with 0.1 μmol/L spironolactone (S3378; MilliporeSigma) or vehicle before 8 hours of exposure to DM conditions or mannitol (MilliporeSigma) (osmotic) control prior to mRNA harvest and cDNA conversion using standard techniques. Gene expression was determined using TaqMan Gene Expression Assays (Applied Biosystems) for *MMP2* (Hs01548727_m1), *MMP9* (Hs00957562_m1), and *MMP14* (Hs01037003_g1). The 2^–ΔΔCT^ method of quantification was used to calculate the fold change, normalized to *GAPDH* (Hs02786624_g1). To study cell surface changes cells were pretreated with spironolactone or vehicle for 24 hours prior to 6 days exposure to DM conditions or osmotic control media, respectively. At the experimental end, cells were fixed (4% PFA) but not permeabilized prior to blocking with 1% BSA in PBS for 30 minutes and following a standard immunofluorescence protocol. Primary antibodies or WGA lectin were incubated overnight at 4°C (MMP2, 10373-2, Proteintech, 1:100; MMP9, AF909, R&D Systems, 1:100; MMP14, EP1264Y, Abcam, 1:1,000; WGA, FITC-WGA, GTX01502, GeneTex, 5 mg/mL, 1:500). FITC-labeled secondary antibodies were incubated at room temperature for 2 hours. Negative controls were processed in the absence of the primary antibody and revealed no staining. The cell surface was imaged as previously using standardized settings. Florescence intensity was measured in randomly selected microscope fields (mean florescence intensity for cell covered area minus background intensity) by an observer in a blinded manner.

### Human renal samples.

Renal biopsy samples from 34 patients were obtained from the Histopathology Department of Southmead Hospital (Bristol, United Kingdom) and Aldo Moro University of Bari (Division of Nephrology, Dialysis and Transplantation, Department of Emergency and Organ Transplantation; Bari, Italy). Tissue was immersion fixed in 10% neutral buffered formalin and embedded in paraffin. Samples had been clinicopathologically diagnosed, including 19 patients with DN, 8 patients with TBMN and 7 histologically normal control patients (Table 1). TBMN is the most common cause of persistent hematuria, and patients usually display minimal or no proteinuria, normal renal function, and a uniformly thinned GBM (107). TBMN occurs in more than 1% of the population and is considered a lifelong nonprogressive disorder (108). With limited numbers of histologically normal renal core biopsy samples available, we utilized samples from patients with TBMN to provide an additional comparison group.

### Human urine samples.

Urine samples from 35 patients were obtained from a double-blind, placebo-controlled trial conducted in patients with diabetes as previously described (57). Briefly, the study population consisted of male and female subjects with type 1 or type 2 diabetes mellitus who satisfied the following criteria: (a) seated systolic BP > 130 mmHg and albuminuria (uACR ≥ 300 mg/g); (b) all patients received the ACEi lisinopril (80 mg/day) and were randomly assigned to placebo or spironolactone (25 mg/day) for the 48 weeks; and (c) urine samples were obtained for baseline and 48-week measurements, from 20 placebo patients and 15 spironolactone patients (Table 2).

### Statistics.

All statistics were calculated using Prism 8 (GraphPad). Normality was assessed visually and using the Shapiro-Wilk test. Where data was normally distributed, a 1-way ANOVA or Student’s 2-tailed *t* test was conducted. If data were not normally distributed, the nonparametric equivalents of these tests were used (Kruskal-Wallis 1-way ANOVA on ranks or Mann-Whitney rank-sum test) to establish if data were significant. The Tukey method (1-way ANOVA) or Dunn’s method (ranks) was used for multiple comparisons between groups. All data are expressed as mean ± SEM unless stated otherwise. Results with values of *P* < 0.05 were considered statistically significant. Individual rats were considered experimental units within this study.

### Study approval.

All animal protocols were approved by the UK Government Home Office and conformed to the *Guide for the Care and Use of Laboratory Animals* (National Academies Press, 2011). All human studies were approved by national and local research ethics committees (REC) and conducted in accordance with the tenets of the Declaration of Helsinki. Human renal samples provided from Bristol were archived anonymized samples (REC H0102/45). For renal samples provided from Bari, Italy, patients gave written informed consent for the use of this material for research purposes (Prot. N.4104/2013). Human urine samples were obtained from a double-blind, placebo-controlled trial conducted in patients with diabetes; this study was approved by the UT Southwestern Medical Center IRB (ClinicalTrials.gov; NCT00381134).

## Author contributions

MC, GIW, RRF, SCS, and MJB designed research; MC, JKF, RDR, ASO, LKD, and MJB performed research; JS, CJD, SJH, PP, LG, HLH, and RDT contributed resources; MC, JKF, KLO, ASO, MG, LS, KHW, and MJB analyzed data; MC, SCS, and MJB wrote paper; and all authors approved the final version of the manuscript.

## Supplementary Material

Supplemental data

## Figures and Tables

**Table 1:**
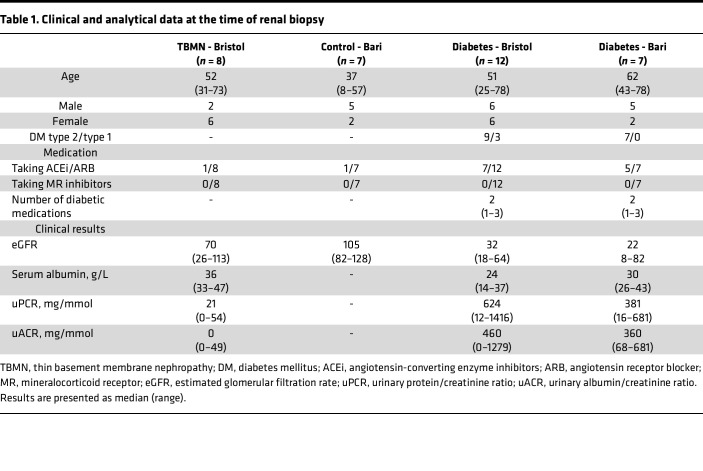
Clinical and analytical data at the time of renal biopsy

**Table 2:**
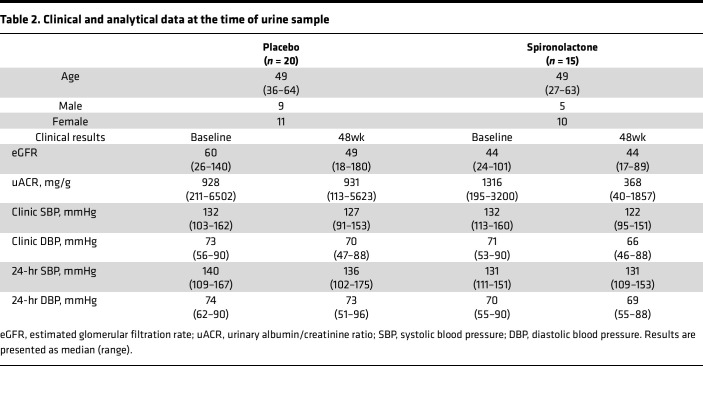
Clinical and analytical data at the time of urine sample

## Version 1 02/07/2023

In-Press Preview

## Version 2 03/08/2023

Electronic publication
