# Supplementary material for: Mineralocorticoid receptor antagonism in diabetes reduces albuminuria by preserving the glomerular endothelial glycocalyx
Source: JCI Insight. 2023 Mar 8;8(5):e154164. doi: 10.1172/jci.insight.154164 (PMC10077489; doi:10.1172/jci.insight.154164)
Supplement: Supplemental data [file jciinsight-8-154164-s198.pdf]

Supplementary Material for

Mineralocorticoid receptor antagonism in diabetes reduces albuminuria by preserving the glomerular endothelial glycocalyx

Michael Crompton, Joanne K. Ferguson, Raina D. Ramnath, Karen L. Onions, Anna S. Ogier, Monica Gamez, Colin J. Down, Laura Skinner, Kitty H. Wong, Lauren K. Dixon, Judit Sutak, Steven J. Harper, Paola Pontrelli, Loreto Gesualdo, Hiddo L. Heerspink, Robert D. Toto, Gavin I. Welsh, Rebecca R. Foster, Simon C. Satchell, Matthew J. Butler

Corresponding Author: Matthew J. Butler
Address: Bristol Renal, Dorothy Hodgkin Building, Whitson St, Bristol, BS1 3NY, UK
Telephone: +44 (0)117 331 3086
Email: matthew.butler@bristol.ac.uk

Supplementary Figures

Supplemental Figure 1. Streptozotocin (STZ)-induced rats were hyperglycaemic, development of albuminuria and proteinuria in early diabetic nephropathy were limited by MR antagonism

Supplemental Figure 2. Transmission electron microscopy confirmed that MR antagonism prevented diabetes-induced glomerular endothelial glycocalyx damage.

Supplemental Figure 3. Early diabetic nephropathy was not associated with other endothelial, podocyte or glomerular parameter changes.

Supplemental Figure 4. The effect of infused hyaluronidase is focused on the endothelial glycocalyx, resulting in isolated GEnGlx degradation and no other detectable changes in the filtration barrier.

Supplemental Figure 5. Exposing human GEnC to diabetic conditions resulted in GEnGlx damage and *MMP* mRNA up-regulation that were ameliorated by MR antagonism.

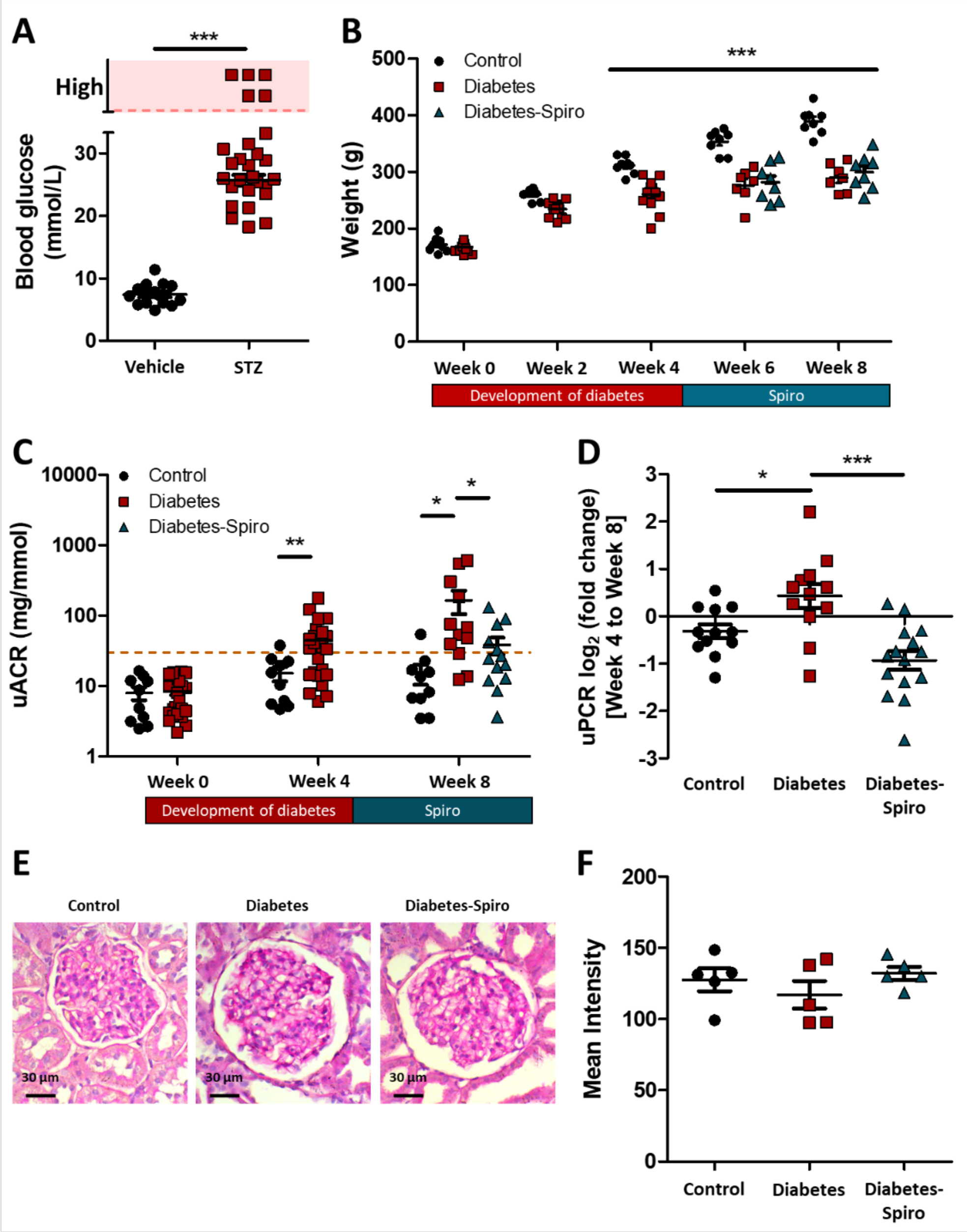

Supplemental Figure 1. Streptozotocin (STZ)-induced rats were hyperglycaemic, development of albuminuria and proteinuria in early diabetic nephropathy were limited by MR antagonism. (**A**) Rats were hyperglycaemic at week 3 post-STZ (vehicle, *n*=14; STZ, *n*=27). Unpaired t test was used for statistical analysis. (**B**) Body weight was not significant between groups when compared at week 0 but from week 4 post-STZ both vehicle and spironolactone (spiro) treated diabetic rats were significantly lower than control rats (control, *n*=8; diabetes, *n*=7; diabetes-spiro, *n*=8). Repeated measures two-way ANNOVA was used for statistical analysis followed by Bonferroni's multiple comparisons. (**C**) Albuminuria levels stabilise in diabetic rats treated with spiro but progressively worsen in rats given vehicle. Urinary albumin:creatinine ratio

(uACR) was determined at week 0, week 4, and week 8 (control, *n*=10; diabetes, *n*=12; diabetes-spiro, *n*=14). A base-10 log scale is used for the Y axis. Repeated measures two-way ANNOVA (Week 0 to Week 4) and one-way ANOVA (Week 8) was used for statistical analysis followed by Bonferroni's and Tukey's multiple comparisons, respectively. (**D**) Urinary protein:creatinine ratio (uPCR) was determined at week 4 and week 8 showing treatment with spiro reduced the fold change in uPCR from initiation of treatment (control, *n*=12; diabetes, *n*=12; diabetes-spiro, *n*=15). Data was log transformed and presented as $\log_2$ (fold change). (**E**) Representative images of glomeruli stained with periodic acid–Schiff (PAS) are shown for control, diabetes, and diabetes-spiro samples. Bars = 30 μm. (**F**) Quantification of PAS staining at week 8 post-STZ show no significant differences. One-way ANOVA was used for statistical analysis. Each dot, triangle, or square on the graphs represent a rat. Data are expressed as mean ± SEM. * $P<0.05$; ** $P<0.01$; *** $P<0.001$.

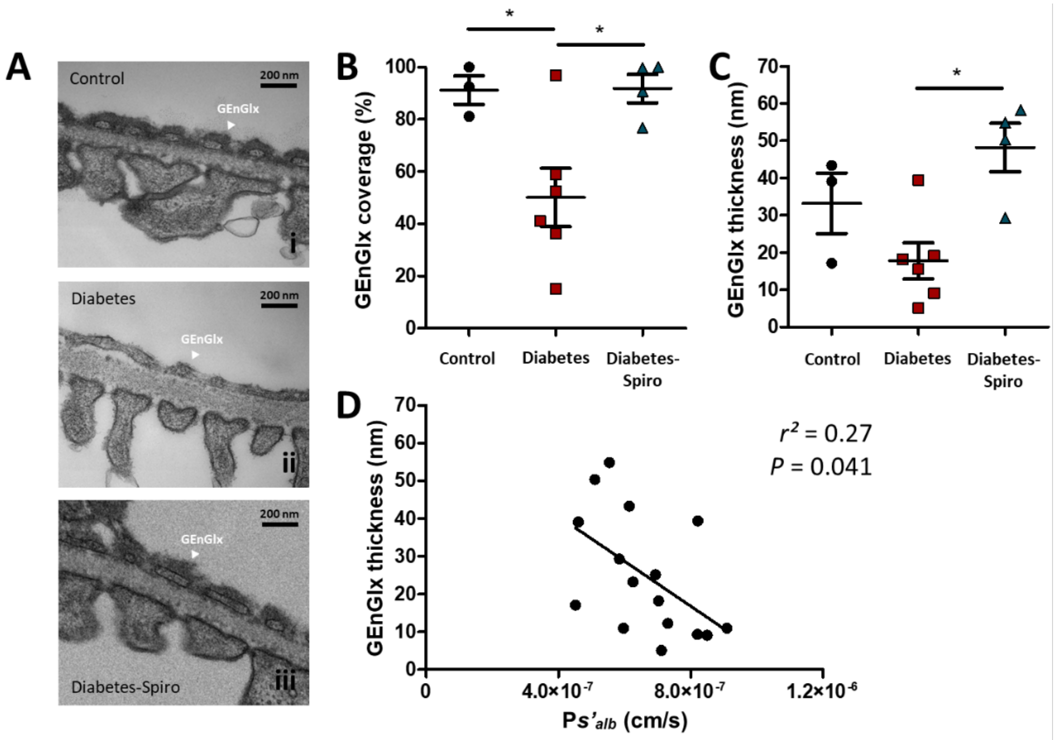

Supplemental Figure 2. Transmission electron microscopy confirmed that MR antagonism prevented diabetes-induced glomerular endothelial glycocalyx damage. Rats were perfusion-fixed for transmission electron microscopy (TEM) with cacodylate buffer containing glutaraldehyde and Alcian blue. (**A**) Representative electron micrographs of the glomerular capillary wall are shown for control, diabetes, and diabetes-spironolactone (spiro) samples. Labels indicate glomerular endothelial glycocalyx (GEnGlx). Bars = 200 nm. Quantification at week 8 post-STZ of (**B**) GEnGlx coverage, and (**C**) GEnGlx thickness (control, *n*=3 rats (9 glomeruli); diabetes, *n*=6(13); diabetes-spiro, *n*=4(8)). In B and C, one-way ANOVA was used for statistical analysis followed by Tukey's multiple comparisons. (**D**) The rate of glomerular albumin leakage (Ps'_{alb}) is weakly associated with GEnGlx thickness measured by TEM (*n*=16). Each dot, triangle, and square on the graph represents a rat. Data are expressed as mean ± SEM. * P<0.05.

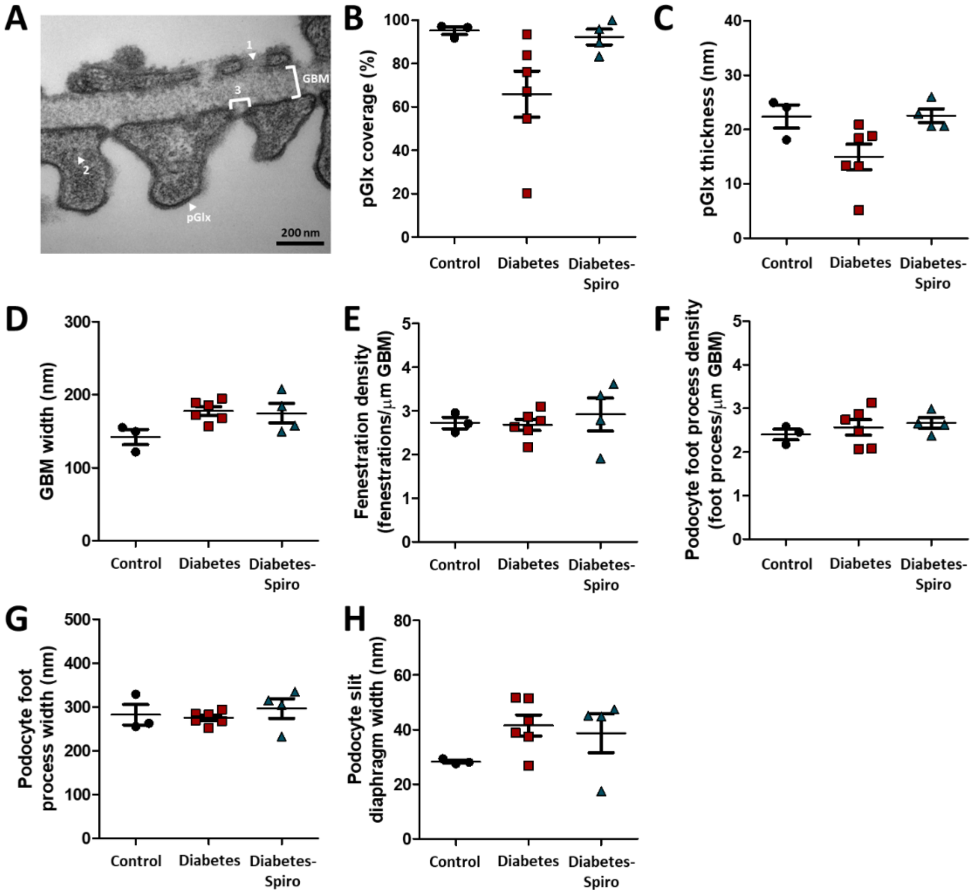

Supplemental Figure 3. Early diabetic nephropathy was not associated with other endothelial, podocyte or glomerular parameter changes. Rats were perfusion-fixed for transmission electron microscopy with cacodylate buffer containing glutaraldehyde and Alcian blue. (**A**) Representative electron micrographs of the glomerular capillary wall with labels indicating podocyte glycocalyx (pGlx), glomerular basement membrane (GBM), (1) fenestration, (2) podocyte foot process, and (3) slit diaphragm. Bar = 200 nm. Quantification at week 8 post-STZ of (**B**) pGlx coverage, (**C**) pGlx thickness, (**D**) GBM width, (**E**) fenestration density, (**F**) podocyte foot process density, (**G**) podocyte foot process width, and (**H**) slit diaphragm width (control, *n*=3 rats (9 glomeruli); diabetes, *n*=6(13); diabetes-spiro, *n*=4(8)). In B-H, one-way ANOVA was used for statistical analysis. Each dot, triangle, or square on the graph represents a rat. Data are expressed as mean ± SEM.

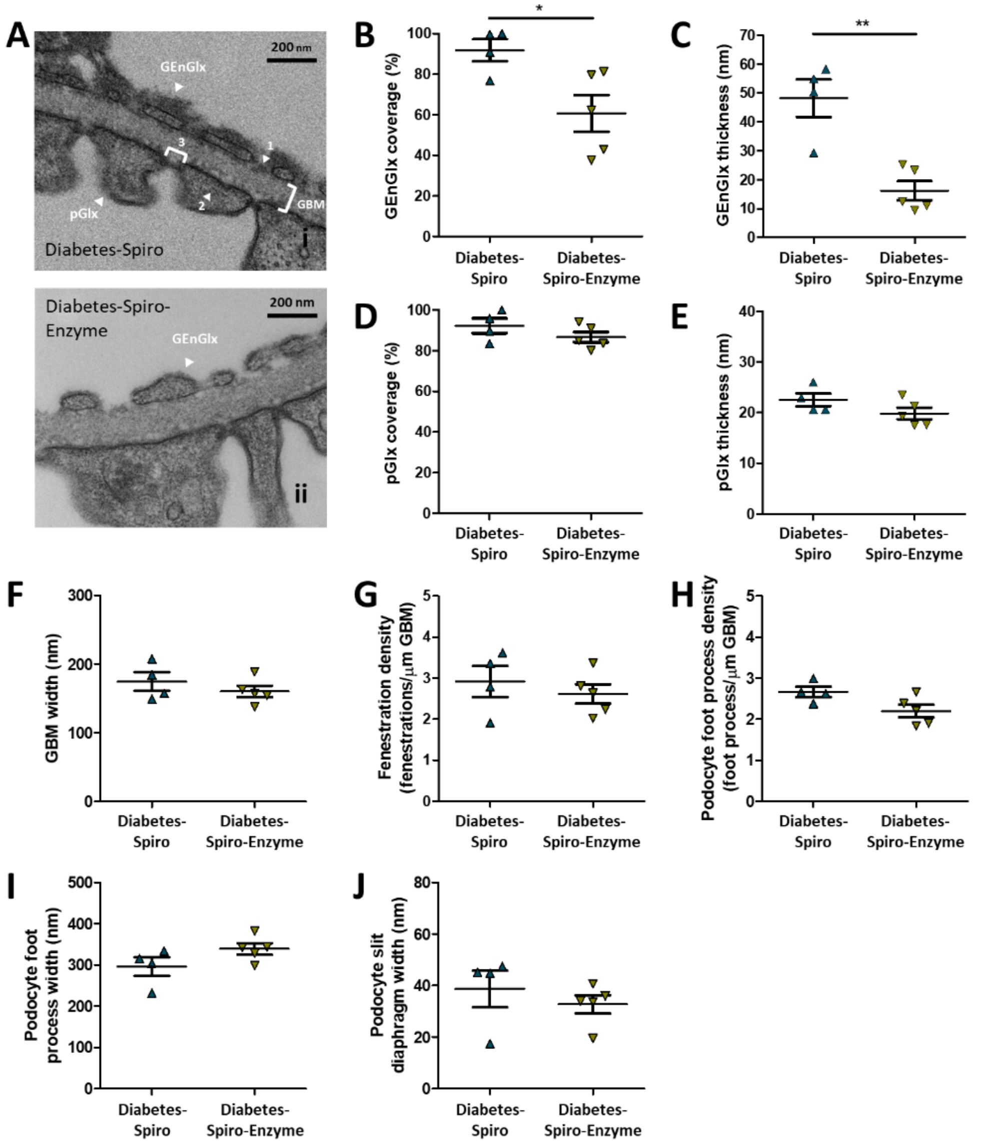

Supplemental Figure 4. The effect of infused hyaluronidase is focused on the endothelial glycocalyx, resulting in isolated GEnGlx degradation and no other detectable changes in the filtration barrier. Rats were perfusion-fixed for transmission electron microscopy with cacodylate buffer containing glutaraldehyde and Alcian blue. (**A**) Representative electron micrographs of the glomerular capillary wall are shown for diabetes-spiro, and diabetes-spiro-enzyme samples. Labels indicate glomerular endothelial glycocalyx (GEnGlx), podocyte glycocalyx (pGlx), glomerular basement membrane (GBM), (1) fenestration, (2) podocyte foot process, and (3) slit diaphragm. Bars = 200 nm. TEM quantification at week 8 post-STZ of (**B**) GEnGlx coverage, and (**C**) GEnGlx thickness (diabetes-spironolactone (spiro), *n*=4 rats (8 glomeruli); diabetes-spiro-enzyme, *n*=5(14)) confirmed enzyme degradation of GEnGlx. Quantification at week 8 post-STZ of (**D**) pGlx coverage, (**E**) pGlx thickness, (**F**) GBM width, (**G**) fenestration density, (**H**) podocyte foot

process density, (**I**) podocyte foot process width, and (**J**) slit diaphragm width. In B-J, unpaired t test was used for statistical analysis Each triangle on the graph represents a rat. Data are expressed as mean ± SEM. * $P<0.05$; ** $P<0.01$.

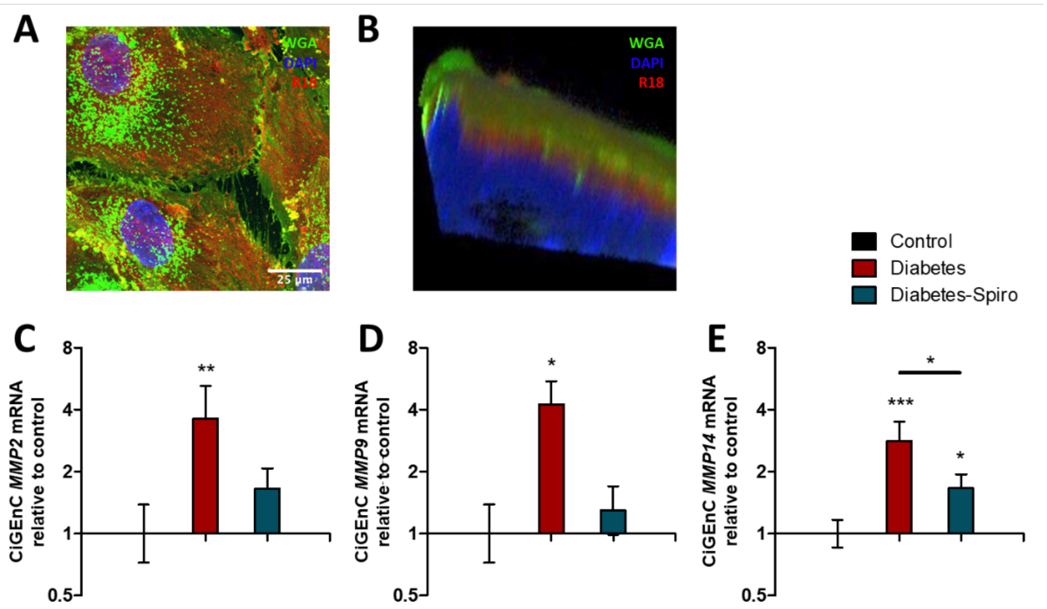

Supplemental Figure 5. Exposing human GEnC to diabetic conditions resulted in GEnGlx damage and *MMP* mRNA up-regulation that were ameliorated by MR antagonism. Human conditionally immortalized glomerular endothelial cells (CiGEnC) maintained in the presence of glucose, insulin, TNF-α and IL-6 to mimic a diabetic environment. Representative images of CiGEnC control samples stained with WGA lectin showing (**A**) 3D projection of Z-stack images and (B) 3D volume rendering with a sliced view showing endothelial glycocalyx stained with WGA lectin. DAPI, nuclear label; R18, membrane label. Bar = 25 µm. Gene expression in CiGEnC was determined using TaqMan RT-qPCR for (**C**) *MMP2*, (**D**) *MMP9*, and (**E**) *MMP14*. The $2^{-\Delta\Delta CT}$ method of quantification was used to calculate the fold change, normalized to *GAPDH* (*n*=6 per group). In C-E, one-way ANOVA was used for statistical analysis followed by Tukey's multiple comparisons. Data are expressed as mean ± SEM. * P<0.05; ** P<0.01; *** P<0.001.
